# Effect of Several *Naja atra* Antivenom Injection Methods on the Rabbit Model of *Naja naja atra* Bite Poisoning

**DOI:** 10.1155/2023/3253771

**Published:** 2023-02-20

**Authors:** Jie Yang, Jin-Cheng Li, Zhou Huang, Dong-Ling Huang, Fan Wang, Wan-Xia Wei, Ji-Fei Nong, Feng Yang, Xue-Ling Lu, Jun-Rong Zhu, Wei Wang

**Affiliations:** Department of Emergency, The First Affiliated Hospital, Guangxi Medical University, Nanning, Guangxi, China

## Abstract

Snakebite is a global public health concern, which often occurs in tropical and subtropical underdeveloped areas, but it is often neglected. In the southern China, *Naja naja atra* (*Chinese cobra*) is a common venomous snake that causes swelling and necrosis of local tissues, even amputation and death. Currently, the main therapy is the administration of *Naja atra* antivenom, which greatly reduces mortality. However, the antivenom is not particularly effective in the improvement of local tissue necrosis. Clinically, antivenom is mainly administered intravenously. We speculated that the method of injection influences the efficacy of antivenom. In this study, the rabbit model was used to explore the effects of different antivenom injection methods on systemic and local poisoning symptoms. If topical injection of antivenom contributes to ameliorate tissue necrosis, then we need to reconsider the use of *Naja atra* antivenom.

## 1. Introduction

People in rural areas of underdeveloped countries are at considerable risk of venomous snakebites of tropical and subtropical species, particularly in south Asia, where such instances are frequent [1]. Up to now, more than 3000 species of snakes are known to exist in the world, about 15% of which are lethal, and more than 60 species of venomous snakes have been discovered in China [2, 3]. Despite the high fatality and disability rates linked with poisonous snakebites, these tropical diseases are not addressed seriously [4]. According to epidemiological statistics, between 1.8 and 2.7 million individuals worldwide are bitten by venomous snakes each year, of which approximately 100,000 die, and more than 400,000 are disfigured or permanently crippled as a result [5]. Humans have an intrinsic fear of snakes, causing them to occasionally develop psychiatric issues after being bitten by venomous snakes [6, 7].

*Naja naja atra* (*N. atra*) is a member of the *Elapidae* family, one of the 10 most poisonous snakes in China, which primarily lives in the south of the Yangtze River. *N. atra* snakebites account for approximately 17% of all snakebites each year; hence, *N. atra* is recognized as a venomous snake of high medical importance in China. Venomous snakes are divided into four groups based on how their venom affects an organism: neurotoxic, hemotoxic, cytotoxic, and mixed venom. *N. atra* is a cytotoxic venomous snake. Clinical manifestations of *N. atra* poisoning include varying degrees of tissue swelling and necrosis surrounding the bite site, which in severe cases can spread throughout the torso. Unlike other members of the *Elapidae* family, despite the presence of neurotoxin in the venom of *N. atra*, the patient shows no signs of coma or respiratory paralysis [8–11].

The widely known antidote to snakebite toxicity is antivenom [12]. In China, the principal therapy for *N. atra* bite poisoning is antivenom administered intravenously. Although the rate of deaths and multiple organ dysfunction syndromes has decreased, no influence on the development of local tissue necrosis appears to have occurred [10]. Despite early antivenom administration and even increasing the dose, the necrosis worsens [13]. Tissue necrosis warrants additional study as a severe consequence of *N. atra* snakebite poisoning. Most of the earlier investigations focused on the efficacy of various antivenoms in neutralizing the toxin, with few addressing changes in serum biochemistry before and after antivenom treatment, as well as the influence of antivenom injection methods on systemic and local symptoms. We hypothesized that topical antivenom administration would efficiently infiltrate the bite region and then quickly neutralize the toxin, reducing the necrotic area and preventing further worsening of tissue necrosis. As a result, we used rabbit as the experimental animal in this study to investigate the effects of different antivenom injection methods on systemic and local poisoning symptoms.

## 2. Materials and Methods

### 2.1. Animal Group

Thirty-five healthy growing adult New Zealand white rabbits (2.5–3.0 kg) supplied by the Experimental Animal Center (Guangxi Medical University, China) were housed in a housing facility where rabbit chow and water could be provided. Animals were acclimatized in the housing facility for a week before the experiment began. Rabbits were randomly assigned to five groups (*n* = 7): (1) blank control group, saline injection only (A group); (2) model group, venom injection only (B group); (3) intravenous injection group (C group); (4) subcutaneous injection group (D group); and (5) in situ injection group (E group). The animal experiment protocol was reviewed and approved by the First Affiliated Hospital of Guangxi Medical University Ethical Review Committee, China (approval number: 2022-KY-E-(227), July 7, 2022).

### 2.2. Venom and Antivenom

Guangxi Snake Venom Research Institute donated lyophilized *N. atra* crude venom, which was maintained at −20°C until utilized. *Naja atra* antivenom (1000 IU, 10 ml, batch no. 20201202) was purchased from Sailun Biotechnology Co., Ltd. The antivenom, a form of monospecific horse anticobra venom immunoglobulin that is degraded by gastric enzymes, was stored in a light resistant refrigerator at 4°C until it was used. Two vials (2000 IU) of antivenom can neutralize the amount of venom discharged by *N. atra*.

### 2.3. Establishment of Animal Model

Through preexperiment, it was found that the *N. atra* venom solution made from 0.466 mg/kg venom lyophilized powder and 8 *μ*l/kg sterile saline could induce necrosis in the muscle tissue, and the model established with this solution was stable. After weighing the rabbits, their right hindlimb hair was shaved, and they were secured to the operation table. Next, a microinjector was used to inject the venom solution, at a dose of 8.0 *μ*l/kg of body weight, into the muscle of the right hindlimb at a depth of approximately 3 mm. Except for the A group and the B group, each group was treated accordingly. 60 minutes after being envenomed, the rabbits of the C group were intravenously injected, via the auricular vein, with 12 IU/kg of antivenom diluted in 3 ml/kg sterile saline; the rabbits of the D group were subcutaneously injected, to form a “ring” about 1cm in diameter around the site of the venom injected, with 12 IU/kg of antivenom diluted in 1ml/kg sterile saline, and the rabbits of the E group received an in situ intramuscular injection, at the site where venom was injected, of 12 IU/kg of antivenom, diluted in 0.3 ml/kg sterile saline solution. All rabbits injected with venom were administered local analgesia containing 2% lidocaine hydrochloride and 0.1% epinephrine.

### 2.4. Serum Biochemistry Examination

Blood samples (2-3 ml) from the auricular artery of five groups were collected 2, 6, and 12 hours after envenomation for biochemistry testing. The drawn blood was allowed to stand at room temperature for 30 minutes before being centrifuged for 10 minutes at 3,500rpm to separate the serum for various biochemistry tests. The separated serum was used for testing the activity of aspartate aminotransferase (AST), alanine aminotransferase (ALT), serum urea nitrogen (BUN), creatinine (Cr), creatine kinase (CK), and creatine kinase isoenzyme MB (CK-MB). Those projects were measured using commercial kits (manufactured by Rayto Life Sciences Co., Ltd., China).

### 2.5. Measurement of the Necrotic Area

The key criterion for determining myonecrosis was the necrotic area of the damaged muscle. Three days after the venom injection, animals were sacrificed using an excessive pentobarbital solution. The right hindlimb was dissected, and the necrotic tissue was entirely separated. The length of the necrotic area's major and minor axes was measured with a ruler, and the size of the figure was computed using a mathematical formula.

### 2.6. Histological Examination

Following necrotic area measurement, the necrotic tissue was promptly rinsed with saline and immersed in a 4% paraformaldehyde solution for 24 hours before being dehydrated and embedded in paraffin. The paraffin block was sliced into sections, and the 3 *μ*m sections were dewaxed and stained with hematoxylin and eosin before pathological examination with a light microscope (Eclipse E100, Nikon, Japan) and digital camera.

### 2.7. Statistical Analysis

The data of this study were analyzed using SPSS 22.0 statistical software. The measurement data of normal distribution are expressed as mean ± standard error of the mean (SEM), and the *t*-test of independent samples is used for comparison between the two groups. ANOVA was used for comparison among three or more groups. LSD *t*-test was used for pairwise comparison between the groups, with *p* < 0.05 indicating statistical significance for the aforementioned statistical tests. The error bars of the graph represent the standard error of the mean.

## 3. Results

### 3.1. Changes in Biochemical Indices after the Antivenom Injection

Table 1 displays the changes in biochemical indices after 2 hours following the injection of *N. atra* crude venom. The serum AST, BUN, Cr, and CK-MB levels of each group were not significantly different from those of the A group; however, the levels of ALT in the model, D, and E groups were greater than those of the A group. The serum CK level of each group was higher than that of the A group.

Table 2 displays the variations in the levels of biochemical indices 6 hours after the venom injection. The levels of AST increased significantly in the B, D, and E groups, whereas the levels of ALT remained higher in these groups than those in the A group. In addition, the release of serum BUN and Cr was greater in the B and the C groups than that in the A group. The serum CK-MB of the C group remained the same as that of the A group and was lower than that of the D and E groups. Although the levels of serum CK in each group increased significantly, the levels in the E group were lower than those in the B group.

Table 3 displays the variations in the levels of biochemical indices 12 hours after the venom injection. Serum AST and ALT levels reduced in the B, D, and E groups but remained higher than in the A group. Except for serum BUN, Cr, and CK, the levels of all indices in the C group were not significantly different from the A group. The serum BUN and Cr levels in the D and E groups were not significantly different from the A group. Although the levels of CK in the D and E groups remained higher than those in the A group, they decreased significantly when compared to the B group, and the level in the E group was lower than that in the D group.

### 3.2. Local Blockage and In Situ Injections with Antivenom Reduced the Necrotic Area

**Figure 1:**
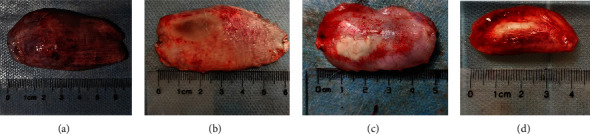
Necrotic lesions in the muscle of the hindlimbs of envenomed rabbits (photographs taken by Xue-Ling Lu and Jun-Rong Zhu).

**Figure 2:**
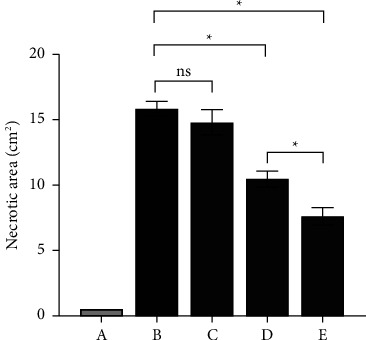
Necrotic area in different groups. The necrotic areas are represented with mean and SEM. There was no significant difference between the B and C groups, *p* > 0.05. The necrotic areas of the D and E groups were less than those of the model group, with the E group having the smallest area, *p* < 0.05. The unit of necrotic area is square centimeter (cm^2^).

As shown in Figure 1, the necrotic tissue dissected was oval in shape, the necrotic area was milky white, and the hyperemia area was red. After being figured out with a ruler, the following is a short summary of the experiment's results: 15.82 ± 0.57 cm^2^ in the B group, 14.81 ± 0.92 cm^2^ in the C group, 10.46 ± 0.60 cm^2^ in the D group, and 7.6 ± 0.63 cm^2^ in the E group (Figure 2). The difference in the necrotic area between the B group and the D and E groups was statistically significant (*p* < 0.05). Furthermore, the necrotic area of the D group was greater than that of the E group (*p* < 0.05). As a result of this section, we discovered that both subcutaneous and in situ intramuscular injections had the effect of confining the necrotic area, which was consistent with a reduction in serum CK, although the latter was more evident.

### 3.3. HE Staining

**Figure 3:**
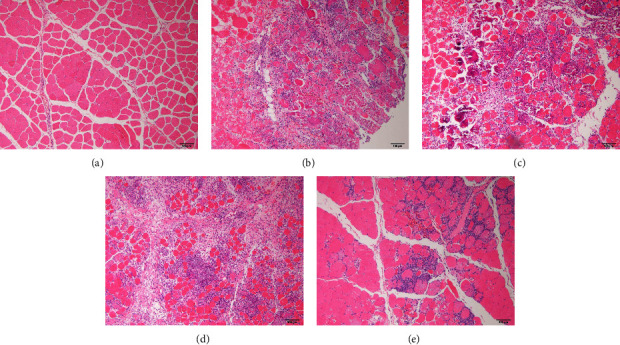
Histological investigation of the tissue necrosis. (a) A group; (b) B group; (c) C group; (d) D group; (e) E group. Bars = 100 *μ*m.

Using HE staining, the necrotic tissue of rabbits was detected.  Figure 3 demonstrates that the myocytes of the A group (panel a) were polygonal with distinct boundaries, and their fibers were organized regularly and closely within fascicles. The muscle damage in the B (panel b), C (panel c), and D groups (panel d) was severe, with indistinct muscle boundaries, varied muscle fiber and diameters, and even fragmentation due to cell membrane damage. A substantial number of inflammatory cells also invaded the necrotic region. In contrast, the structure of fascicles was more intact in the E group (panel e), in which the fascicle was not totally obliterated, myofibers were only minimally injured by *N. atra* venom, and the majority of cells retained their usual morphology. Although there was inflammatory infiltration between normal cells in the E group, there were not as many inflammatory cells there as in the B group. These findings also suggested that in situ antivenom injections may have a protective impact on the affected tissue.

## 4. Discussion

The use of antivenom improves the patient's systemic poisoning symptoms, but local tissue necrosis caused by *N. atra* bite poisoning remains a common and difficult complication. Cytotoxin (also known as cardiotoxin) is the primary toxin responsible for tissue necrosis and irreversible injury [14]. To address the issue that intravenous antivenom injections do not improve local necrosis, we proposed that local antivenom injections can quickly block the effect of cytotoxin and evaluated the effect of local injections on alleviating the symptoms of systemic poisoning. Thus, in a rabbit model, this study evaluated the relieving effect of antivenom on systemic and local signs of rabbits using three different injection methods.

Snake venoms are mixtures of different protein families. *Elapid* venoms are dominated by three finger toxin (3-FTx) and phospholipase A_2_ (PLA_2_) [15]. In addition to a small amount of nerve growth factor, cysteine-rich secretory protein, and snake venom metalloproteinase, 3-FTx and PLA_2_ made up the majority, 84.3% and 12.2%, respectively, of the *N. atra* venom proteome [11]. Cytotoxin belongs to 3-FTx, which accounts for approximately 55% of freeze-dried crude *N. atra* venom, with the cytotoxic activity leading to tissue necrosis and cardiotoxic activity causing heart injury. The degree of tissue necrosis is correlated with the dose of cytotoxin [16–19].

In the current investigation, the increase in serum AST and ALT levels in poisoned rabbits could be attributed to hepatocyte injury. Although the cause of *N. atra *venom-induced liver injury is unknown, PLA_2_ appears to be involved in hepatocyte destruction. PLA_2_ inhibited the activity of Na^+^/K^+^ ATPase, causing a lipid bilayer disruption in the plasma membrane which resulted in hepatocyte apoptosis [20]. Furthermore, rats exposed to crude venom showed a considerable increase in hepatic oxidative stress markers, suggesting that the venom has a harmful effect on the liver by causing oxidative stress [21].

The cardiac damage caused by *N. atra* bite poisoning is induced by cytotoxin, and cardiac arrest may occur in severe cases. As antivenom has become more widespread, the incidence of cardiac arrest has reduced. After injections of snake venom into rabbits, the release of serum CK-MB, one of the sensitive markers of myocardial injury, increased over time. Some researchers believe that the PLA_2_ in the venom of the subspecies also has cytotoxicity and can lead to cell necrosis [22, 23]. It should be noted that PLA_2_ with myotoxic activity is primarily basic in nature; the alkaline charge of PLA_2_ is essential for binding to cell membranes and damaging cells [22]. Actually, the PLA_2_, as purified from the venom of *N. atra*, belongs to the acidic subtype, which has an Asp residue at position 49 and lacks myotoxic activity [11]. However, Asp49 PLA_2_ enhances the cytotoxic activity and, through a synergistic interaction with cytotoxin, causes severe tissue necrosis [24].

Venoms of *Viperidae* family and *Elapidae* may be nephrotoxic, leading to acute kidney injury [25]. According to the findings of our investigation, venom-injected rabbits released considerably more serum BUN and Cr than healthy rabbits did. Contrary to expectations, blood BUN and Cr did not drop after an intravenous dose of antivenom but did reduce with in situ injections, indicating that *N. atra* venom does not directly react with renal tissues.

According to pharmacokinetic study data, cytotoxin can be absorbed into the systemic circulation and rapidly reach its absorption peak (30 minutes), but its intramuscular bioavailability is poor, only 45.6%, indicating that a significant amount of cytotoxin remains at the injection site [26]. Cytotoxins, on the other hand, produce microvessel thrombosis by increasing platelet aggregation triggered by adenosine diphosphate, thrombin, collagen, or PLA_2_ [27]. The formation of microvascular thrombosis leads to tissue ischemia and hypoxia at the bite site, which is not conducive to the arrival of antivenom to the bite site. In this investigation, the intravenous injection of antivenom was unable to alleviate local tissue necrosis, but in situ injection reduced the necrotic area and serum levels of BUN and Cr, suggesting that aberrant renal function was caused by necrosis of muscle tissues following *N. atra* poisoning. As a result, when necrosis was reduced, renal function was restored. The issue with topical antivenom injections is delayed and inadequate absorption, and the beneficial effect on heart and liver damage was not as noticeable as that of intravenous administration [28].

Snake venom, as a complex mixture, has a direct effect on local and systemic symptoms, but the immune response it induces results in the release of inflammatory mediators (TNF-*α*, IL-1, IL-6, and IL-10), which deteriorate cell and tissue damage [29]. Unfortunately, antivenom is incapable of neutralizing these mediators. Furthermore, the cytotoxin of *N. atra* venom has a high affinity for local tissues and causes the lysis of muscle membranes in a short period of time [26, 30].

## 5. Conclusions

Our findings reveal that antivenom administered intravenously was effective in neutralizing toxins that had entered the systemic circulation and in recovering cardiac and hepatic damage but did not ameliorate the necrotic area. In situ antivenom injections did not improve cardiac and liver damage as compared to intravenous administration, but it did limit the development of tissue necrosis and restore renal function. The difference in improving local tissue necrosis between intravenous injections and in situ injections suggests that we should have a new knowledge of antivenom use, and victims poisoned by *N. atra* snakebite augmenting with in situ injections of antivenom may achieve improved efficacy.

## Figures and Tables

**Table 1:** Outcomes of blood biochemical analysis in rabbits 2 hours after the injection with *Naja atra* venom.

	2 hours				
	*A*	*B*	*C*	*D*	*E*
AST (U/L)	29.71 ± 2.78	33.71 ± 3.15	30.29 ± 2.91	32.43 ± 2.70	26.29 ± 2.83
ALT (U/L)	35.71 ± 3.39	54.71 ± 5.29^*∗*^	36.57 ± 4.89a	66.86 ± 4.82^*∗*^	53.57 ± 4.79^*∗*^
BUN (mmol/L)	5.53 ± 0.48	5.33 ± 0.51	5.09 ± 0.38	5.19 ± 0.29	4.36 ± 0.43
Cr (*μ*mol/L)	49.43 ± 4.24	50.14 ± 3.14	44.71 ± 3.82	47.92 ± 2.09	45.71 ± 2.24
CK (U/L)	1025.43 ± 130.49	3390.29 ± 368.63^*∗*^	3517.57 ± 177.92^*∗*^	3943 ± 248.08^*∗*^	3745.71 ± 203.56^*∗*^
CK-MB (U/L)	360.14 ± 50.25	392.71 ± 45.50	381 ± 19.51	423.86 ± 36.74	413.71 ± 27.72

Values are represented as mean ± SEM (*n* = 7). The asterisk in the columns represents a significant difference with respect to the blank control group, when *p* < 0.05. The letter in the columns represents a significant difference with respect to the model group, when *p* < 0.05.

**Table 2:** Outcomes of blood biochemical analysis in rabbits 6 hours after the injection with *Naja atra* venom.

Parameters	6 h				
	*A*	*B*	*C*	*D*	*E*
AST (U/L)	29.71 ± 2.78	56.71 ± 7.31^*∗*^	28.86 ± 3.13a	51.43 ± 2.50^*∗*^	57.57 ± 5.56^*∗*^
ALT (U/L)	35.71 ± 3.39	67.43 ± 4.58^*∗*^	36.71 ± 4.12a	66.86 ± 3.94^*∗*^	60.29 ± 4.39^*∗*^
BUN (mmol/L)	5.53 ± 0.48	7.16 ± 0.42^*∗*^	7.23 ± 0.35^*∗*^	5.35 ± 0.67a	5.08 ± 0.41a
Cr (*μ*mol/L)	49.43 ± 4.24	69 ± 4.35^*∗*^	66.57 ± 4.54^*∗*^	51.43 ± 2.42a	50.71 ± 5.11a
CK (U/L)	1025.43 ± 130.49	5798.71 ± 396.25^*∗*^	5840.29 ± 306.23^*∗*^	5627.29 ± 157.43^*∗*^	4393 ± 265.30^*∗*^a
CK-MB (U/L)	360.14 ± 50.25	1038.86 ± 68.36^*∗*^	382.71 ± 40.39a	1058.71 ± 82.19^*∗*^	953.57 ± 71.28^*∗*^

Values are represented as mean ± SEM (*n* = 7). The asterisk in the columns represents a significant difference with respect to the blank control group, when *p* < 0.05. The letter in the columns represents a significant difference with respect to the model group, when *p* < 0.05.

**Table 3:** Outcomes of blood biochemical analysis in rabbits 12 hours after the injection with *Naja atra* venom.

Parameters	12 h				
	*A*	*B*	*C*	*D*	*E*
AST (U/L)	29.71 ± 2.78	50.71 ± 3.61^*∗*^	29.14 ± 3.28a	44.55 ± 5.31^*∗*^	51.43 ± 5.44^*∗*^
ALT (U/L)	35.71 ± 3.39	58.57 ± 6.02^*∗*^	35.86 ± 3.64a	55.57 ± 3.56^*∗*^	55 ± 4.38^*∗*^
BUN (mmol/L)	5.53 ± 0.48	7.10 ± 0.53^*∗*^	7.03 ± 0.20^*∗*^	5.64 ± 0.51a	5.61 ± 0.30a
Cr (*μ*mol/L)	49.43 ± 4.24	68 ± 2.52^*∗*^	66 ± 2.90^*∗*^	48 ± 3.49a	49.71 ± 3.75a
CK (U/L)	1025.43 ± 130.49	6421.43 ± 413.28^*∗*^	6352.86 ± 298.93^*∗*^	5105 ± 352.51^*∗*^a	4114.43 ± 231.74^*∗*^a
CK-MB (U/L)	360.14 ± 50.25	2162 ± 128.41^*∗*^	498.57 ± 51.81a	1594 ± 52.41^*∗*^	1564.43 ± 136.67^*∗*^

Values are represented as mean ± SEM (*n* = 7). The asterisk in the columns represents a significant difference with respect to the blank control group, when *p* < 0.05. The letter in the columns represents a significant difference with respect to the model group, when *p* < 0.05.

## Data Availability

The data used to support the findings of this study are included within the article.

## Abbreviations

3-FTx: Three finger toxin
ALT: Alanine aminotransferase
AST: Aspartate aminotransferase
BUN: Serum urea nitrogen
CK: Creatine kinase
CK-MB: Creatine kinase isoenzyme MB
Cr: Creatinine
*N. atra*: *Naja naja atra*
PLA2: Phospholipase A2
SEM: Standard error of the mean.
